# Extracellular Vesicle Secretion from 3D Culture of Human Adipose-Derived Mesenchymal Stem Cells in Scalable Bioreactors

**DOI:** 10.3390/bioengineering12090933

**Published:** 2025-08-29

**Authors:** Shaoyang Ma, Justice Ene, Colton McGarraugh, Shaoxuan Ma, Colin Esmonde, Yuan Liu, Yan Li

**Affiliations:** 1 Department of Chemical and Biomedical Engineering, FAMU-FSU College of Engineering, 2525 Pottsdamer St., Tallahassee, FL 32310, USA or yanghohhot@gmail.com (S.M.); je17d@fsu.edu (J.E.); cmcgarraugh@fsu.edu (C.M.); lilyma782@gmail.com (S.M.); cme18b@fsu.edu (C.E.); 2 Division of Biology and Medicine, Brown University, 69 Brown St., Providence, RI 02912, USA; 3 Lawton Chiles High School, 7200 Lawton Chiles Ln, Tallahassee, FL 32312, USA

**Keywords:** human mesenchymal stem cells, aggregates, vertical wheel bioreactor, extracellular vesicles, biogenesis, neural degeneration

## Abstract

Human mesenchymal stem cells (hMSCs) and their secreted extracellular vesicles (EVs) are promising therapeutics to treat degenerative or inflammatory diseases such as ischemic stroke and Alzheimer’s disease (AD). hMSC-EVs have the coveted ability to contain therapeutically relevant biomaterials; however, EV biogenesis is sensitive to the culture microenvironment in vitro. Recently, the demand for hMSC-EVs has increased dramatically, highlighting the need for scalable bioreactors for large-scale biomanufacturing. In this study, adipose-derived hMSCs were seeded in 2D plates, an ultralow-attachment (ULA) plates as static aggregates, a novel vertical wheel bioreactor (VWBR) as aggregates, and a spinner flask bioreactor (SFB). EV secretion was quantified and compared using ExtraPEG-based ultracentrifugation and nanoparticle tracking analysis. Compared to the 2D group, significantly higher total EV production and cell productivity in the bioreactors were observed, as well as the upregulation of EV biogenesis genes. Furthermore, there was increased EV production in the VWBR compared to the SFB and the static ULA control. Functional assessments demonstrated that EVs, when delivered via culture medium or hydrogel-based systems, significantly attenuated oxidative stress elevation, suppressed proinflammatory cytokine secretion (e.g., TNF-α) and gene expression, and inhibited nuclear factor kappa-light-chain-enhancer of activated B-cell (NF-κB) activation and neurodegenerative markers across in vitro assays. These findings suggest EV-mediated mitigation of oxidative and inflammatory pathways, potentially through modulation of the NF-κB signaling cascade. This study shows the influence of bioreactor types and their microenvironments on EV secretion in hMSCs and their applications in hMSC-EV production and bioengineering.

## 1. Introduction

Human mesenchymal stem cells (hMSCs) are a group of adult stem cells that are derived from a variety of tissues from the mesodermal germ layer such as bone marrow, adipose tissue, and umbilical cord [1]. These cells have emerged as a possible therapeutic treatment for numerous inflammatory, autoimmune, and degenerative diseases, including rheumatoid arthritis (NCT03691909) [2], cystic fibrosis (NCT02866721) [3], Alzheimer’s (NCT03367403) [4], stroke (NCT03356821) [5], host vs. graft (NCT00366145) [6], and cancer [7], due to their self-renewal, multilineage differentiation, and immunomodulation capacities [8,9]. hMSCs fundamentally differentiate into trilineages of osteoblasts, adipocytes, and chondrocytes [10], but these mesoderm-derived stem cells have been reported for multipotent differentiation potential into tissues such as cartilage, bone, muscle, liver, nerve, and myocardium under modulated in vitro conditions [11]. Despite their differentiation potential, a lack of migration capacity at lesion sites post-transplantation suggests another “mode of action” for hMSC therapeutic effects, with recent research suggesting that the therapeutic effects of hMSCs can be linked to their immunoregulatory and pro-regenerative secretome through paracrine factors [9,12,13]. Contained within the cell secretome, extracellular vesicles (EVs) and their small (30–200 nm) subset, exosomes, have been suggested as an integral mechanism in tissue repair capabilities, with preclinical reports showing the effectiveness of EVs in the treatment of non-healing wounds [12,13]. These EVs are part of cellular communication systems and contain multiple therapeutically relevant macromolecules such as lipids, proteins, and nucleic acids [14,15,16]. The clinically significant properties of EVs and their exosome subset have led to an increase in demand for hMSCs and their derived EVs. This surge in demand has necessitated the employment of novel bioreactor systems capable of large-scale production of these biological molecules while preserving their intrinsic properties [17]. Biomanufacturing capacity is one of the main challenges for stem cells and their derived EVs for therapeutic applications [18], as it is necessary to obtain up to tens to hundreds of millions of cells of the secreted EVs per patient in a typical clinical dose [19].

Currently, the demand for a large EV quantity has led to a variety of strategies being explored for the generation of EVs from cells. Common strategies include genetic modification [20], cell culture methods [21], and various priming choices [21,22,23]. Three-dimensional culture systems, especially those utilizing bioreactors, have shown significant advancement, as these systems enable dramatic scaling up from 2D culture, as well as provide more precise control, allowing better recapitulation of the in vivo niche [9]. Some influences on cell expansion are cell-to-cell interactions, physical forces, and cell adhesion surface factors, which are variable in different culture systems [24,25]. However, there is limited knowledge on how the bioreactor environment influences EV biogenesis, especially with a major focus on the dynamic microenvironment in contrast with static 2D cultures, which have large differences in fluid dynamics, nutrient transfer kinetics, shear stress, oxidation, and aggregation. These changes can cause the alteration and even enhancement of cellular proliferation, differentiation, and secretome properties, all impacting the therapeutic potential of hMSCs [26,27]. Among these stimuli, recent studies have shown that mechanical stimuli such as shear stress, cyclic stretch, and compression can promote hMSC EV production [28,29]. Proposed mechanisms include the activation of the calcium induction mechanism involved in EV biogenesis [30], while shear stress has also been linked with piezoreceptor activation [31,32]. Despite these early insights, the influences on specific mechanical effects on EV biogenesis and its pathways are incompletely understood. Furthermore, hMSC expansion as aggregates or on microcarriers in bioreactors has a seminal impact on the biological and therapeutic properties of hMSCs, such as increasing cytokine secretion and differentiation ability [33,34,35]. Although recent studies have shown additional possible mechanisms relating the dynamic microenvironments of bioreactors to cell properties such as metabolism and senescence [36,37], the exact mechanisms that govern hMSC properties, such as EV secretion under dynamic microenvironments, are not completely understood.

In this study, it was hypothesized that the biophysical microenvironment profiles present in the dynamic 3D culture of bioreactors alter EV production, increasing the amount of EVs secreted in bioreactors compared to static cultures. Furthermore, it was hypothesized that the different shear stress profiles present in vertical wheel bioreactor (VWBR) and spinner flask bioreactor (SFB) systems have varying effects on EV biogenesis in hMSCs, characterized through EV production. Previous studies have shown the successful expansion of hMSCs and induced pluripotent stem cells (iPSCs) in various bioreactor systems, but focus has not been heavily placed on the production of EVs, an integral mechanism managing the therapeutic effect of hMSCs [38,39,40]. In our previous studies, bone marrow-derived hMSCs were found to increase EV secretion in SFB and VWBR systems on microcarriers and as aggregates in wave motion [41,42], and VWBRs have also been used to generate EVs from human iPSCs [43,44]. This would be the first proof-of-concept study to grow adipose derived hMSC aggregates in a VWBR.

This study provides a direct comparison between the two types of scalable bioreactors as well as between dynamic and static cultures for EV biogenesis. The comparison was performed with microcarrier-based seeding in the SFB, as hMSC aggregates cannot be formed in an SFB based on our previous experiments. A static hMSC aggregate control was also used to elucidate the influence of VWBR hydrodynamics under the same 3D culture organization. Furthermore, functional testing was performed to examine the reduction in oxidative stress and neural inflammation by hMSC-EVs. EVs were shown to reduce neural degeneration in cells treated with conditioned media of brain organoids derived from iPSCs of a sporadic AD patient with an APOE ε4/ε4 mutation that models neural degeneration in Alzheimer’s disease.

## 2. Methods and Materials

### 2.1. Human Mesenchymal Stem Cell Culture

Frozen adipose-derived hMSCs at passage 1 were acquired from the Tulane Center for Stem Cell Research and Regenerative Medicine. The hMSCs were isolated from the subcutaneous abdominal adipose tissue from three deidentified healthy donors that were younger than 45 years with a body mass index lower than 25. These cells were initially seeded in a 5% CO_2_ humidified Forma™ series II water-jacketed CO_2_ incubator (Thermo Fisher Scientific, Waltham, MA, USA). The hMSCs were seeded in 0.22 µm filtered complete medium containing alpha-minimal essential medium (MEM) (Life Technologies, Carlsbad, CA, USA)-based media, 10% fetal bovine serum (FBS) (Gibco, Thermo Fisher Scientific, Waltham, MA, USA), and 1% penicillin–streptomycin (Life Technologies). Regular media exchanges occurred every 2–4 days, with cells being passaged or harvested with a 0.25% trypsin–ethylenediaminetetraacetic acid (EDTA) (Invitrogen, Grand Island, NY, USA) solution. The trypsin–EDTA solution was neutralized with culture media and subsequently centrifuged at 500× *g* for 5 min. The cell pellet was resuspended in media for passaging or seeding.

### 2.2. 2D Culture

**Figure 1:**
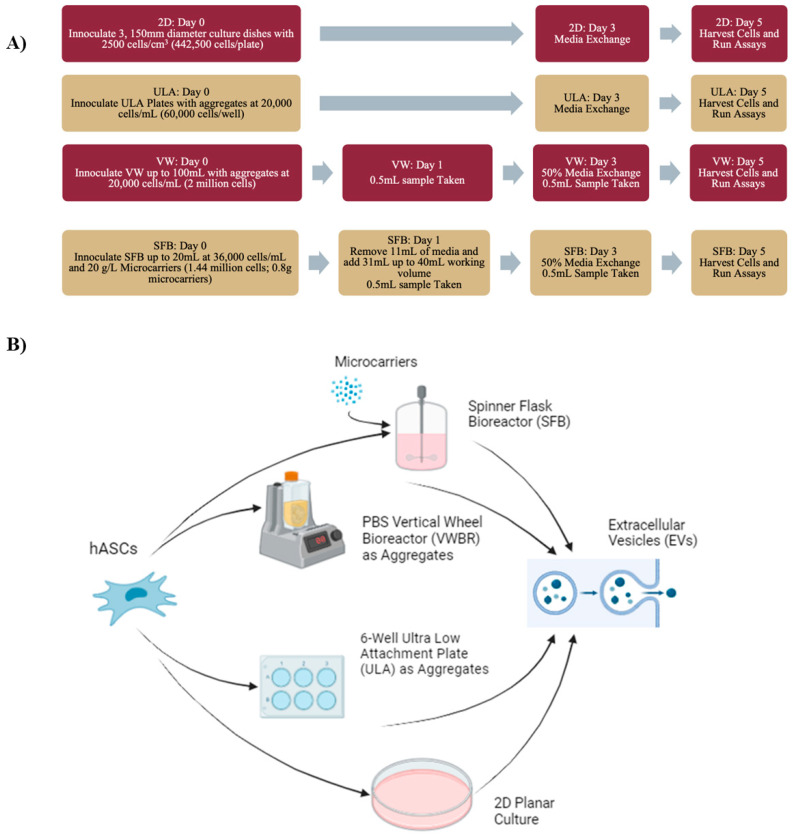
Schematic representations of experimental procedure. (**A**) Flowchart of procedure, along with bioreactor settings, sampling dates, and experimental overview. Synthemax II microcarriers: 30 cm^2^/gram. Total surface area for 40 mL and 0.8 g microcarriers was 288 cm^2^. With 1.44 million cells and 50–70% seeding efficiency for Synthemax II microcarriers, seeding density for an SFB is 2500–3500 cells/cm^2^. With 20,000 cells/mL, aggregates had a seeding density of around 3000–3600 cells/cm^2^ in the VWBR. (**B**) Diagram of experimental bioreactors, the static aggregate control, and 2D control. Depicts hMSC seeding and collected EVs. Image created with BioRender.

The cells in the 2D culture were inoculated at 2500 cell/cm^2^ in 150 mm-diameter plastic petri dishes (Corning, Corning, NY, USA). The 2D subcultures were cultured up to passage 6 before seeding into bioreactor and 2D planar control groups, as illustrated in Figure 1.

As visualized in Figure 1, hMSC expansion was conducted in 4 parallel settings: a 100 mL Wheaton spinner flask bioreactor (SFB), a 100 mL PBS vertical wheel bioreactor (VW), an ultralow-attachment (ULA) 6-well plate (Corning Incorporated, Corning, NY, USA), and a traditional 2D culture. Three plastic petri dishes were seeded at 2500 cells/cm^2^ or 442,500 cells/plate at day 0 and kept at 37 °C and 5% CO_2_ for incubation with 13.5 mL medium/plate. The static hMSC aggregate control was performed in ULA 6-well plates in parallel. Briefly, cells were seeded at 20,000 cells/mL in 3 mL media in each well of ULA 6-well plates. Pictures taken with a microscope (Olympus IX70, Olympus, Melville, NY, USA) were used to record culture morphology on days 1, 3, and 5. Medium changes for the 2D culture and the other culture systems were executed concurrently on day 3 post-seeding. The 2D culture underwent a complete medium replacement, while the other culture systems received a 50% medium exchange. These procedures were carried out in parallel to maintain temporal consistency across all culture systems. The cells were harvested using the trypsin–EDTA solution. The supernatant at day 5 was collected separately for EV isolation.

### 2.3. Spinner Flask Bioreactor Culture

hMSCs were inoculated in a 100 mL Wheaton spinner flask with a working volume of 40 mL. hMSCs were seeded with 50% of the working volume at 36,000 cells/mL (1.44 million total cells) at day 0. Synthemax II microcarriers were added at 20 g/L (0.8 g total). The seeded bioreactor was placed on a Wheaton Biostir magnetic stirrer system (DWK Life Sciences, Millville, NJ, USA). Cycles with an intermittent-agitation seeding method with 50 rpm at 6 min on and 24 min off were used. On day 1, 25% media was removed to discard detached or dead cells, and 75% media was added to raise the total volume to 40 mL. On day 1, the stir settings were changed to the running settings of 50 rpm at 20 min on and 10 min off. On days 1, 3, and 5, 0.5 mL samples were collected, stained with Hoechst 33342 (Thermofisher, Waltham, MA, USA), and viewed under the Olympus IX70 fluorescence microscope to visually inspect cell morphology on the microcarrier surface. A 50% media change was conducted on day 3 concurrently with other culture systems. On day 5, the supernatant was carefully collected for EV isolation without disturbing the cells with microcarriers.

### 2.4. Vertical Wheel Bioreactor Culture

hMSCs were inoculated in a 100 mL PBS vertical wheel bioreactor (PBS Biotech Inc., Camarillo, CA, USA) as aggregates without the use of microcarriers. Cells were added at 20,000 cells/mL to a total of 2 million cells. Media were added directly up to the working volume of 100 mL, and the bioreactor was set to continuous agitation of 60 rpm. On days 1, 3, and 5, 0.5 mL samples were collected, stained with Hoechst 33342 (Thermofisher), and viewed under the Olympus IX70 fluorescence microscope to visually inspect cell morphology. A 50% media change was conducted on day 3 concurrently with other culture systems. On day 5, all cells were collected from the bioreactor and centrifuged into a pellet. The supernatant was collected separately for EV isolation.

### 2.5. Flow Cytometry

Harvested and suspended hMSCs were washed in staining buffer: ice-cold phosphate-buffered saline (PBS) containing 0.5% (wt./vl.) bovine serum albumin (BSA) (Sigma-Aldrich, St. Louis, MO, USA) and fixed with 4% paraformaldehyde (PFA) at room temperature for 15 min. Cells were then permeabilized in 100% methanol. Nonspecific binding sites were blocked in blocking buffer—PBS containing 5% (wt./vl.) BSA—for 30 min at room temperature. Cells were washed with PBS and incubated with anti-Yes-associated protein (YAP) and anti-Sirt-1 primary antibodies (Supplementary Table S1) at room temperature for two hours, followed by one hour’s incubation with the corresponding secondary antibody Alexa Fluor 488 (Supplementary Table S1). Samples were washed in PBS and acquired using a BD FACS Canto II flow cytometer (Becton Dickinson, Franklin Lakes, NJ, USA) along with isotype control. The results were analyzed using FlowJo software (Version 10.10).

### 2.6. Extracellular Vesicle Isolation

For EV isolation, culture media were supplemented with EV-depleted FBS, generated by subjecting standard FBS to ultracentrifugation (UC) using an SW32 rotor at 29,000 rpm (≈120,000× *g*) for 20 h at 4 °C. The supernatant fraction, collected post-centrifugation and filtered through a 0.22 µm membrane, was designated as EV-depleted FBS and utilized to minimize exogenous bovine EV contamination during cell culture.

hMSC EVs were isolated using differential ultracentrifugation. Conditioned media were collected on day 5 and sequentially spun. The media were centrifuged at 500 g for 5 min, 2000 g for 10 min, and 10,000 g for 30 min, with the supernatant being collected and centrifuged again while the pellets were discarded to remove larger vesicles, apoptotic cells, and organelles. After the sequential ultracentrifugation, the collected supernatant was mixed with polyethylene glycol (PEG6000) solution (Sigma-Aldrich) [45], with an end concentration of 8% PEG in 1 M NaCl. The solution was then mixed and stored overnight at 4 °C to enrich EVs. The mixed solution was then centrifuged at 3200× *g* for one hour at 4 °C. The supernatant was discarded with the EV pellets resuspended in 1 mL of PBS and ultracentrifuged at 127,000× *g* for 70 min at 4 °C and 20-micron (equivalent to 2.6 Pa) vacuum in an Optima^TM^ MAX-XP ultracentrifuge (Beckman Coulter, Brea, CA, USA). The resulting pellet was air-dried and resuspended in 100 µL PBS.

### 2.7. Nanoparticle Tracking Analysis (NTA)

NTA was performed on the isolated EV samples in triplicate to determine size distribution and particle concentration. The resuspended EV isolation was further diluted 10-fold with PBS for analysis. NTA was performed on a Nanosight LM10-HS instrument (NTA 3.4 Build 3.4.003, Malvern Instruments, Malvern, UK) configured with a blue (488 nm) laser and CMOS camera. For each replicate, three videos of 60 s were acquired with camera shutter speed fixed at 30.00 ms. To ensure accurate and consistent detection of small particles, the camera level was set to 9 and the detection threshold was maintained at 7. The laser chamber was cleaned thoroughly with particle-free water between each sample reading. The collected videos were analyzed using NTA3.0 software to obtain the mode and mean size distribution, as well as the concentration of particles per milliliter of solution. Compared to the mean size, the mode size is usually a more accurate representation because vesicle aggregates or other particles may skew the value of the mean size.

### 2.8. Transmission Electron Microscopy (TEM)

Electron microscopy imaging was used to confirm the morphology and size of EVs. Briefly, EV isolates were resuspended in 30 μL of filtered PBS. For each sample preparation, intact EVs (15 µL) were dropped onto Parafilm. A carbon-coated 400 Hex mesh copper grid (Electron Microscopy Sciences, EMS, Hatfield, PA, USA) was positioned using forceps with coating side down on top of each drop for 1 h. Grids were rinsed three times with 30 µL filtered PBS before being fixed in 2% PFA for 10 min (EMS, EM Grade). The grids were then transferred on top of a 20 µL drop of 2.5% glutaraldehyde (EMS, EM Grade) and incubated for 10 min. Samples were stained for 10 min with 2% uranyl acetate (EMS grade). Then, the samples were embedded for 10 min with a mixture of 0.13% methyl cellulose and 0.4% uranyl acetate. The coated sides of the grids were left to dry before imaging on an HT7800 transmission electron microscope (Hitachi, Tokio, Japan).

### 2.9. Western Blot

EV and cell samples were lysed in radioimmunoprecipitation assay (RIPA) buffer (150 mM sodium chloride, 1.0% Triton X-100, 0.5% sodium deoxycholate, 0.1% sodium dodecyl sulfate, 50 mM Tris, pH 8, 2 µg/mL aprotinin, 5 µg/mL leupeptin, 5 µg/mL antipain, 1 mM PMSF protease inhibitor) together with 1% of proteinase inhibitor cocktails (Invitrogen). Samples were then digested for 20 min on ice and spun down at 14,000 rpm for 20 min. The supernatant was collected and the protein concentration determined by a Bradford assay. Protein lysate concentrations were normalized, and 20 µg of each sample was denatured at 95 °C in 2 × Laemmli sample buffer. Proteins were loaded into 12% BIS-Tris-SDS gels and transferred onto a nitrocellulose membrane (Bio-rad). The membranes were then blocked for 1 h in 5% skim milk (*w*/*v*) in Tris-buffered saline (10 mM Tris-HCl, pH 7.5, and 150 mM NaCl) with 0.1% Tween 20 (*v*/*v*) (TBST). Membranes were incubated overnight in the presence of the primary antibodies (Supplementary Table S1) diluted in the blocking buffer at 4 °C. Afterward, the membranes were washed four times with TBST for 10 min each time and then incubated with an IR secondary (LI-COR, Lincoln, NE, USA) at 1:5000 for 90 min at room temperature. The blots were then washed four more times with TBST for 10 min each time before being processed with the LI-COR Odyssey (LI-COR Biosciences).

### 2.10. Reverse-Transcription Quantitative Polymerase Chain Reaction (RT-qPCR)

Total RNA was isolated from different cell samples using an RNeasy Mini Kit (Qiagen, Valencia, CA, USA) according to the manufacturer’s protocol. The isolated RNA samples were further treated with a DNA-Free RNA Kit (Zymo, Irvine, CA, USA) to remove genomic DNA contamination. Reverse transcription was carried out according to the manufacturer’s instructions using 2 µg of total RNA, anchored oligo-dT primers (Operon, Huntsville, AL, USA), and Superscript III (Invitrogen, Carlsbad, CA, USA). Oligo Explorer 1.2Primers (Genelink, Hawthorne, NY, USA) software was used to design the real-time PCR primers specific to target genes (Supplementary Table S2). For normalization of expression levels, β-actin was used as an endogenous control. Using SYBR1 Green PCR Master Mix (Applied Biosystems, Foster City, CA, USA), real-time PCR reactions were performed on an ABI7500 instrument (Applied Biosystems). The amplification reactions were performed as follows: 2 min at 50 °C, 10 min at 95 °C, and 40 cycles of 95 °C for 15 s and 55 °C for 30 s, and 68 °C for 30 s following a melt curve analysis. The Ct values of the target genes were first normalized to the Ct values of the endogenous control β-actin. The corrected Ct values were then compared for the bioreactor conditions to the static control. Fold changes in gene expression were calculated using the comparative Ct method, $2^{{-(∆C}_{t\;treatment}-{∆C}_{t\;control})}$, to obtain the relative expression levels.

### 2.11. In Vitro Functional Assay Under Aβ42 Oligomer Treatment

Amyloid beta 1–42 (Aβ42) oligomers were used to mimic AD in vitro. To prepare oligomers of Aβ42 peptide, biotinylated Aβ(1–42) (Bachem) was fully dissolved at 0.5 mg/mL in hexafluor-2-propanole (HFIP, Sigma-Aldrich) [46,47]. HFIP Aβ(1–42) solution (10 μL) was dispensed into a siliconized Snap-Cap microtube, put in a desiccator to completely evaporate HFIP, and thereafter stored at −80 °C. Oligomer solutions were prepared freshly for each experiment. The stock was dissolved in 10 μL of DMSO (to 105 μM) and incubated for 3 h at room temperature. Oligomers of Aβ(1–42) were added to the hMSCs at 1 μM for three days in the absence or presence of the EVs (at the concentration of 1 × 10^8^ EVs/mL). The cultures were evaluated for cytokine secretion and reactive oxygen species (ROS). Briefly, after washing, the cells were treated with 25 μM carboxy-H2DCFDA (Invitrogen). After 30 min incubation at 37 °C (protected from light), the cells were washed and imaged under the fluorescence microscope (Olympus IX70, Melville, NY, USA). ImageJ (Version: 2.16.0/1.54p) analysis was performed on the images (*n* = 5) for ROS intensity.

### 2.12. Enzyme-Linked Immunosorbent Assay (ELISA)

An ELISA was employed for determining the secreted cytokine tumor necrosis factor (TNF)-α. Concentrations of cytokines were measured according to the manufacturers’ instructions (BioLegend, San Diego, CA, USA). Briefly, the capture antibodies were incubated in 96-well microplates overnight at 4 °C. The next day, non-specific binding was blocked for one hour at room temperature, then the samples and standards were added and incubated overnight at 4 °C. Then, detection antibodies were added and incubated at room temperature for one hour. Next, avidin–horseradish peroxidase (HRP) solution was added for 30 min, followed by 3,3′,5,5′-tetramethylbenzidine (TMB) substrate solution for 15 min. Then the reaction was stopped by the stop solution. The absorbance was measured using a microplate reader (Bio-Rad, Richmond, CA, USA) at a wavelength of 450 nm. All cytokine samples were run in triplicate.

### 2.13. In Vitro Functional Assay Under AD Brain Organoid-Conditioned Media Treatment

Differentiated human iPSC-derived pericytes (iPCs) were cultured in DMEM/F12 B27 serum-free medium on Matrigel-coated 12-well plates (seeding density: 0.2 × 10^6^ cells/well). To begin the assay, a media change was conducted, with two groups of 3 wells each replacing the culture medium with AD organoid (from human iPSCs of a sporadic AD patient, MC0020, female, 71.3 years old, *APOE* ε4/ε4 [48])-conditioned media. EVs (10,000 EVs/cell) were also added to 2 plates, creating control (−E4E4−EV), +E4E4−EV, −E4E4+EV, and +E4E4+EV wells. Cells were incubated in the conditioned media for 3 days to assess the impact of AD-associated factors on cellular stress response. Afterward, the cells were washed with PBS and treated with 25 μM carboxy-H2DCFDA (Invitrogen) to measure intracellular reactive ROS levels. Cells were incubated for 30 min at 37 °C while being protected from light. After incubation, the cells were washed with PBS 3 times and resuspended in PBS before the fluorescence was measured by a microplate reader (BioRad Laboratories, Hercules, CA, USA) to assess ROS intensity. Images were also taken with a fluorescence microscope (Zeiss, Oberkochen, Germany). RT-qPCR was also performed as described to evaluate molecular recovery in samples. Alzheimer’s disease-specific markers (APP, BACE1, MAPT, and P53) were assessed in addition to pro- (TNF-α, IL-6, and IL12β) and anti-inflammatory (CD163, IL10, and TGF-β1) markers.

### 2.14. EV Delivery in Hydrogels to Reduce Inflammation

EV encapsulation in collagen hydrogels was used to treat lipopolysaccharide (LPS)-stimulated 3T3 fibroblasts. The 3T3 fibroblast cells (ATCC) were seeded in plates with coverslips coated with fibronectin. Around 30,000 cells were added to each well of 24-well plates in Dulbecco’s modified Eagle’s medium (DMEM) plus 10% FBS and 1% penicillin–streptomycin. The cells were treated with LPS (100 ng/mL, Sigma-Aldrich) for 24 h and then introduced to EVs and collagen–EVs. The collagen hydrogel matrix was prepared using mouse-derived collagen (Sigma-Aldrich), sterile PBS, sterile water, sodium hydroxide, and EVs. The hydrogel was formed by mixing 2 mL of collagen, 0.4 mL of PBS, 46 µL of sodium hydroxide, and 1.5 mL of water. To prepare a total volume of 4 mL hydrogel encapsulated with 1.5 × 10^7^ EVs per well, 46 µL of EV solution with appropriate EV concentration was incorporated into the mixture. After thorough mixing, 0.5 mL of hydrogel was dispensed into each well and incubated overnight at 37 °C to allow complete gelation.

A collagen hydrogel group not infused with EVs was also exposed to the inflamed cells in order to analyze any differences in anti-inflammatory reactions with the infused collagen group. The collagen hydrogel was made similar to the infused group, but due to the lack of volume from the lack of EV concentration mixed in, a total amount of 0.44 mL of PBS was used to make up for the difference. This group was placed on top of the inflamed cells directly after inflammation.

Immunocytochemistry: The samples were fixed with 4% PFA for 1 h, permeabilized with 100% cold methanol, and then were blocked with blocking buffer (5% FBS in PBS) for 30 min. After that, the samples were incubated with primary antibody NF-kB (Supplementary Table S1) at 4 °C overnight and then were stained with the secondary antibody Alexa Fluor^®^ 647 goat anti-rabbit IgG (1:1000, Invitrogen) for 1 h at room temperature. To observe the nuclei, the samples were stained with DAPI (1:2000, Invitrogen) for 3 min at room temperature. The samples were imaged using the fluorescence microscope (Olympus IX70, Melville, NY, USA). The images were converted to .tiff files and uploaded to MATLAB (Version R2022b) for analysis of NF-kB localization in nuclei (indicating inflammation) or cytoplasm.

### 2.15. Statistical Analysis

Experimental results are expressed as means ± standard deviation (SD). Statistical comparisons were performed using ANOVA for multiple comparisons, and significance was set at *p* < 0.05. For direct comparison between two conditions, statistical comparisons were performed with Student’s *t*-test.

## 3. Results

### 3.1. Expansion and Characterization of hMSCs

**Figure 2:**
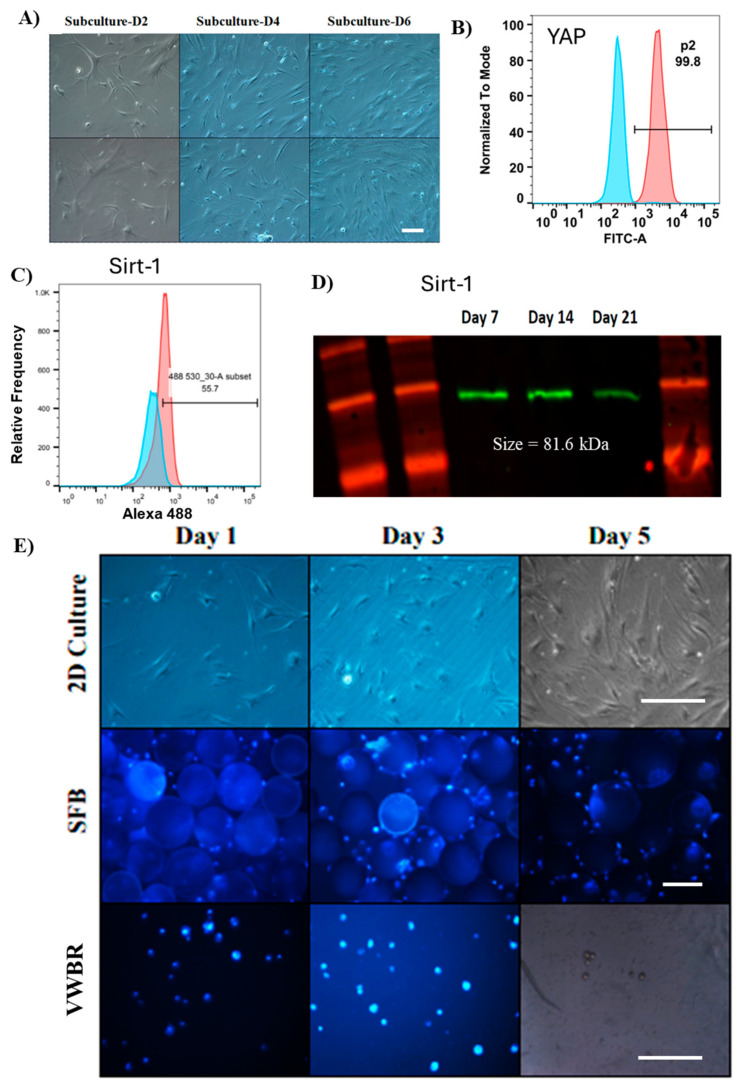
Adipose-derived hMSC expansion and characterization. (**A**) Morphology shows healthy cell culture throughout the subculture before seeding in bioreactors at passage 6. Subculture D2, 4 days before seeding; D4, 2 days before seeding; D6, right before seeding. Scale bar: 50 µm. (**B**) Flow cytometry histogram for the cells stained by YAP expression. (**C**) Flow cytometry histogram of Sirt-1 expression; (**D**) Western blot of Sirt-1 expression, a cellular marker related to senescence. (**E**) Representative imaging of cells taken over the culture period with DAPI staining for 2D and bioreactor runs. Scale bar: 100 µm.

hMSCs were subcultured in planar 2D culture up to passage 6 before seeding into bioreactors. As seen in Figure 2A, constant cell growth was observed consistently, with passaging near confluence. The hMSCs were stained with YAP antibody, and nearly all subcultured cells expressed YAP, as illustrated in Figure 2B, a signaling pathway that regulates cell self-renewal, proliferation, and membrane regeneration [49,50], various hallmarks of a viable stem cell. For EV biogenesis, CD47-enriched EVs have been reported to be released in a YAP-dependent manner [51]. Stiff extracellular matrices (ECMs) have also been reported to promote exosome secretion in a YAP/TAZ pathway-dependent manner [52]. The cells also expressed a high level of Sirt-1 (Figure 2C), a regulator of cellular homeostasis and connector between central energy metabolism to cellular senescence and longevity [53]. The Sirt-1 expression decreased with the culture time, indicating that the cells were within the lifespan and suitable for the experiments (Figure 2D).

At passage 6, hMSCs were harvested with the trypsin–EDTA solution and seeded into the dynamic VWBR, SFB, and the static 2D culture. The SFB was inoculated with 3600 cells/mL with 20 g/L of Synthemax II microcarriers, the VWBR was inoculated with 20,000 cells/mL as cell aggregates, and the traditional 2D control culture was inoculated with 2500 cells/cm^2^. hMSCs were seeded at day 0, with the SFB running at seeding settings, only filled up with media to running volume at day 1. Bioreactor samples were nuclear-stained, with staining showing cell growth and density in all bioreactors and 2D groups, as well as aggregation in bioreactor samples, as seen in Figure 2E.

### 3.2. Extracellular Vesicle Secretion

**Figure 3:**
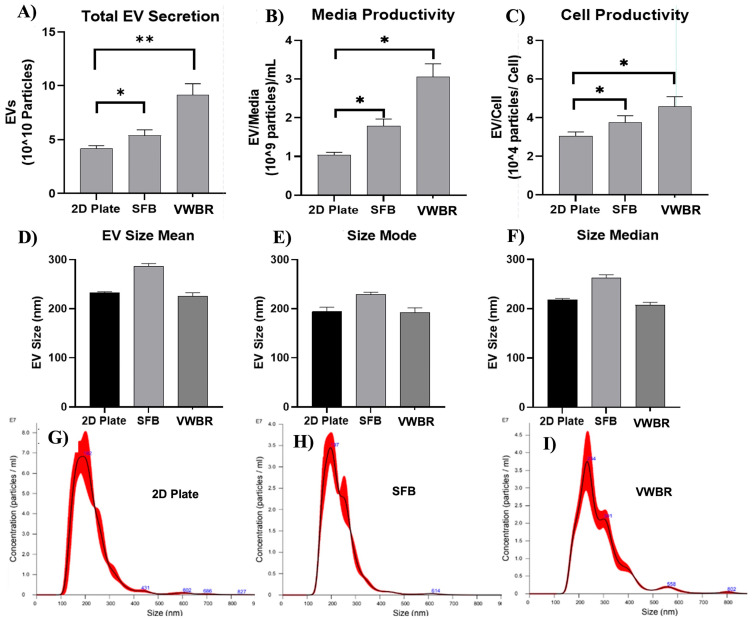
Extracellular vesicle secretion in different culture systems charactered by NTA. (**A**) Total EV yields calculated from NTA. (**B**) EV yields normalized as media productivity (EV particles per mL of media in working volume). Results show a significant increase in media productivity, increasing almost twofold in the SFB group and threefold in the VWBR group. (**C**) EV yields normalized as cell productivity (EV particles per cell count at D0—inoculation). N = 3; * *p* < 0.05, ** *p* < 0.01. EV size as expressed as means + standard deviation. EV size distribution graphs: (**D**) Mean size; (**E**) mode size; (**F**) median size. (**G**) 2D sample; (**H**) SFB; (**I**) VWBR.

The dynamic microenvironment condition in both the VWBR and SFB showed a much higher level of EV production compared to the static 2D culture. The SFB condition exhibited a statistically significant increase in EV secretion, yielding 5.38 × 10^10^ particles compared to 4.15 × 10^10^ particles in the static 2D culture (Figure 3A), representing a 30% increase (*p* < 0.05). Similarly, the VWBR demonstrated a more pronounced and statistically significant enhancement in EV secretion, producing 9.17 × 10^10^ particles. This represents a 121% increase compared to the static 2D culture (Figure 3A, *p* < 0.01).

When the EV count was corrected for media input, the difference was more pronounced than with the total EV count. The 2D group showed 1.04 × 10^9^ particles/mL media compared to the 1.79 × 10^9^ particles/mL of the SFB group, a 74% increase from the static 2D culture, and 3.06 × 10^9^ particles/mL media of the VWBR group, a 194% increase from the static culture and 71% increase from the SFB. Each of the bioreactors also exhibited a statistically significant difference compared to each other (*p* < 0.05) (Figure 3B). When the EV count was corrected for cell number, there was still a difference in EVs/cell produced. The 2D group showed 3.05 × 10^4^ particles/cell compared to the 3.74 × 10^4^ particles/cell of the SFB, a 23% increase from the 2D group, and 4.59 × 10^4^ particles/cell of the VWBR, a 50% increase from the 2D group and 23% for the SFB group. The difference was statistically significant for the EV/media compared to the 2D culture (*p* < 0.05) (Figure 3C). From NTA measurements, graphs for EV mean size (Figure 3A,D) show similar size distributions with slightly larger SFB EVs produced compared to those of the VWBR or 2D groups. For the mode size, similar EV size distributions were observed between 2D and VWBR EVs of 194.0 nm and 191.8 nm, respectively. However, SFB EVs were slightly larger at 225.3 nm (Figure 3B,E). The median size showed a similar trend (Figure 3C,F).

**Figure 4:**
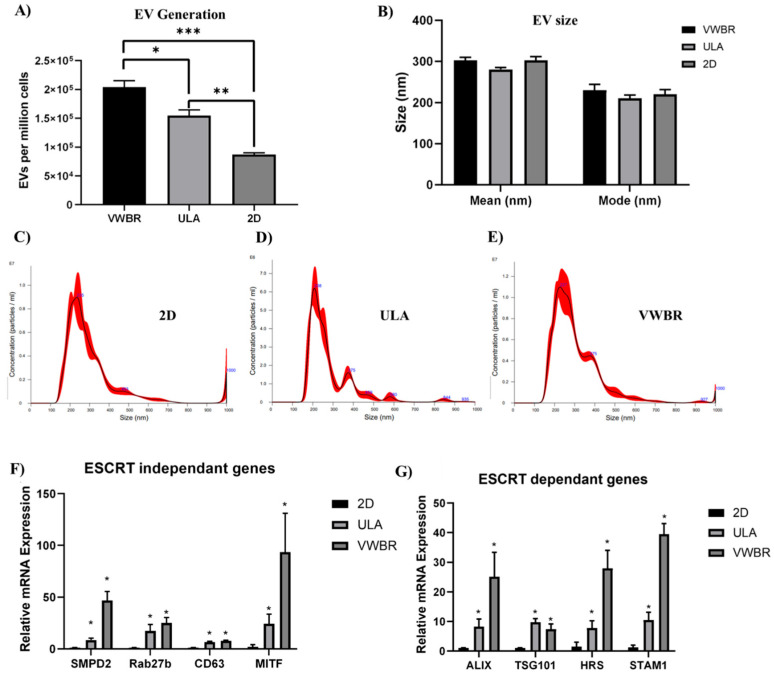
EV biogenesis in VWBR in comparison to static ULA and 2D cultures. (**A**) NTA analysis; (**B**) EV yields normalized as cell productivity (EV particles per cell count at D0—inoculation). EV size as expressed at means + standard deviation. EV size distribution graphs: (**C**) 2D culture; (**D**) static ULA culture; (**E**) vertical wheel bioreactor. EV biogenesis gene expression in the cells compared to the 2D control, determined by RT-qPCR. (**F**) ESCRT-dependent genes; (**G**) ESCRT-independent genes. N = 3; * *p* < 0.05, ** *p* < 0.01, *** *p* < 0.001.

The dynamic microenvironment condition in the VWBR and aggregate formation in the ULA showed a much higher level of EV generation per million cells compared to the static 2D culture. The ULA condition yielded a statistically significant increase in EV secretion at 1.55 × 10^5^ particles per million cells compared to the static 2D culture, with 0.87 × 10^5^ particles (Figure 4A), a 178% (*p* < 0.001) increase. The VWBR also showed a statistically significant increase in EV secretion with 2.04 × 10^5^ particles per million cells compared to the static 2D culture (Figure 4A), with a more pronounced difference at 234% (*p* < 0.0001). Furthermore, the difference between the ULA and VWBR was also statistically significant with a 132% increase (*p* < 0.01). From NTA measurements, graphs for EV mean size (Figure 4B) show similar size distributions, with slightly larger VWBR and 2D EVs produced compared to the ULA group. For the mode size, similar EV size distributions were observed between the VWBR, ULA, and 2D groups, with EVs of 230.5 nm, 210.8, and 220.6 nm, respectively (Figure 4B). From NTA measurements, the distribution of EV particles for all groups is homogeneous, with one prominent peak and some potential smaller peaks at larger sizes, such as in the 2D group (Figure 4C–E).

Gene expression of cells at day 5 was determined. For EV biogenesis in endosomal sorting complex required for transport (ESCRT)-independent and -dependent pathways, ULA (7–24-fold) and VWBR (8–94-fold) groups showed higher expression of all ESCRT-independent genes, such as *SMPD2* (8.5 ± 1.5, 46.8 ± 8.6), *Rab27b* (17.4 ± 6.1, 25.1 ± 5.2), *CD63* (6.7 ± 0.7, 8.0 ± 0.5), and *MITF* (24.3 ± 9.2, 93.6 ± 37.5), than the 2D group (Figure 4F). A similar trend was observed in all ESCRT-dependent genes, with the ULA and VWBR groups showing significantly higher expression in *ALIX* (8.3 ± 2.6, 25.1 ± 8.3), *TSG101* (9.7 ± 1.3, 7.4 ± 1.7), *HRS* (7.8 ± 2.5, 28.0 ± 6.1), and *STAM1* (10.5 ± 2.6, 39.5 ± 3.6) genes, respectively, than the 2D group (Figure 4G). Compared to ULA, the VWBR group showed two- to fourfold higher expression for all the genes except *CD63* and *TSG101*.

### 3.3. Extracellular Vesicle Functional Analysis In Vitro

**Figure 5:**
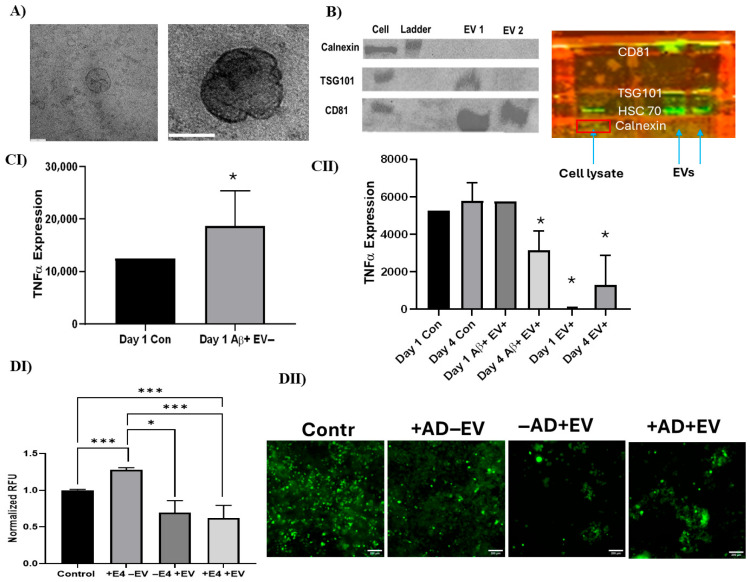
In vitro functional assay of hMSC EVs under Aβ42 oligomer- or AD organoid-conditioned media treatment. (**A**) Transmission electron microscopy (TEM) image of the EVs. Scale bar: 60 nm. (**B**) Western blot of exosomal markers for four different EV samples. (**C**) ELISA analysis for TNF-α secretion. (**I**) Aβ42 oligomers increased TNF-α secretion of the hMSCs; (**II**) EV treatment of Aβ42 oligomer-stimulated cells; (**D**) iPSC-derived pericytes (iPCs) exposed to APOE4 Alzheimer’s disease (AD)-associated brain organoid conditioned media. The effect of hMSC EV treatment on the production of reactive oxygen species (ROS) was evaluated. (**I**) ROS quantification; (**II**) ROS images. Scale bar: 50 µm. * *p* < 0.05; *** *p* < 0.001.

The in vitro function of the hMSC-EVs was evaluated for reduction in oxidative stress and inflammation. The EVs displayed the normal cup shape exosomal morphology, as shown in TEM images (Figure 5A). The vesicles expressed positive exosomal markers CD81, TSG101, and HSC70, and were absent for the negative marker calnexin (Figure 5B). Applying Aβ42 to hMSCs represents an in vitro model to study the effects of Aβ42 on stem cell function and to investigate potential therapeutic approaches for AD. Upon Aβ42 oligomer stimulation, hMSCs showed a high level of ROS production. In the presence of the EVs, the ROS level was reduced to a similar level to the EV only and no treatment controls (Supplementary Figure S1). The secretion of TNF-α was increased upon Aβ42 oligomer stimulation (Figure 5C, Supplementary Figure S2). With EV treatment, the secretion of TNF-α did not show change after day 1, but decreased by 50% after day 4. Notably, the addition of EVs to unstimulated cells also downregulated TNF-α expression at day 1 and day 4, indicating their immunomodulatory capacity (Figure 5C).

**Figure 6:**
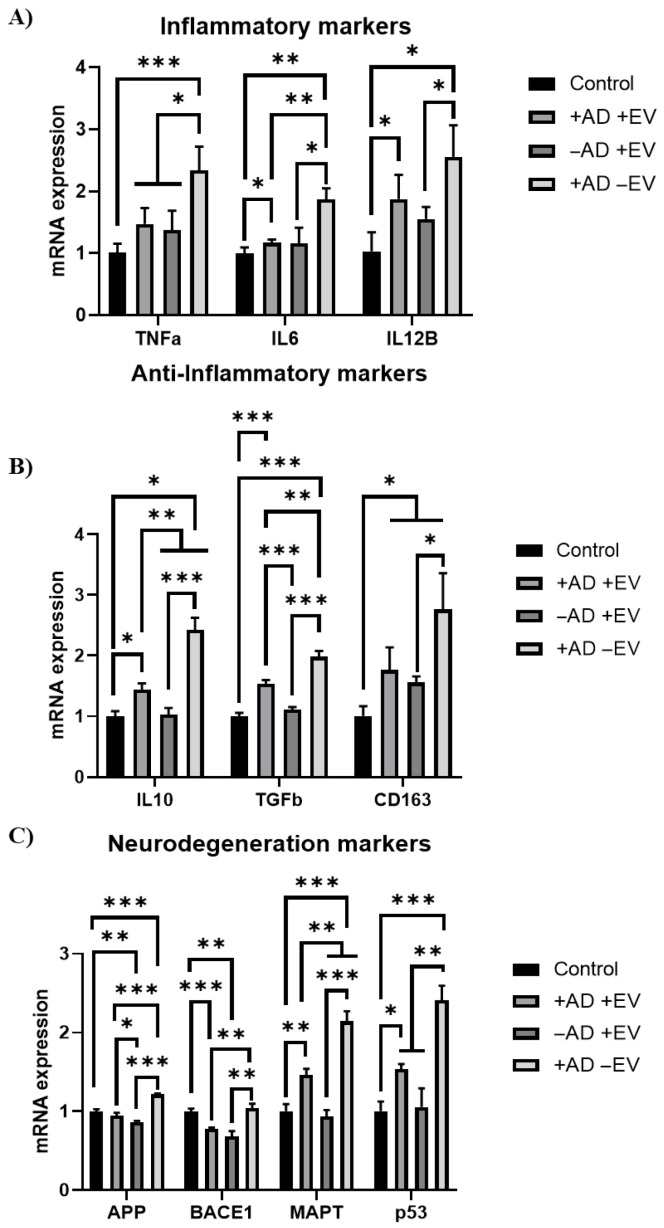
Effects of hMSC EVs on neuroinflammation and neurodegeneration marker expression of iPCs under AD brain organoid-conditioned media treatment. Gene expression was determined by RT-qPCR. (**A**) Inflammation markers; (**B**) anti-inflammatory markers; (**C**) neural degeneration markers. N = 3. * *p* < 0.05; ** *p* < 0.01; *** *p* < 0.001, compared to the control (no AD medium and no EVs).

An in vitro AD model using AD brain organoid-conditioned media was additionally evaluated for cellular recovery after EV treatment (Figure 5D). Among the cells both with and without the addition of AD-conditioned media, the ROS levels of those treated with EVs were all significantly lower than those treated without EVs. Notably, the cells treated with both AD-conditioned media and EVs, in addition to those treated with just EVs, had lower ROS fluorescent intensity than the negative control (without conditioned media or EV treatments). Furthermore, RT-qPCR analysis showed a decrease (around twofold) in mRNA expression of inflammatory markers *TNF-α*, *IL-6*, and *IL12β* in cells cultured with AD conditioned media plus EVs compared to the cells induced with AD-conditioned media (Figure 6A). The expression of anti-inflammatory markers (IL10, TGF-β1, and CD163) also showed similar trends, indicating that the EVs did not increase anti-inflammatory marker expression (Figure 6B). However, EVs were found to decrease (by 20%–67%) the expression of the neurodegenerative markers *APP, BACE1, MAPT*, and *P53* associated with AD compared to the no-EV-treatment group (Figure 6C).

**Figure 7:**
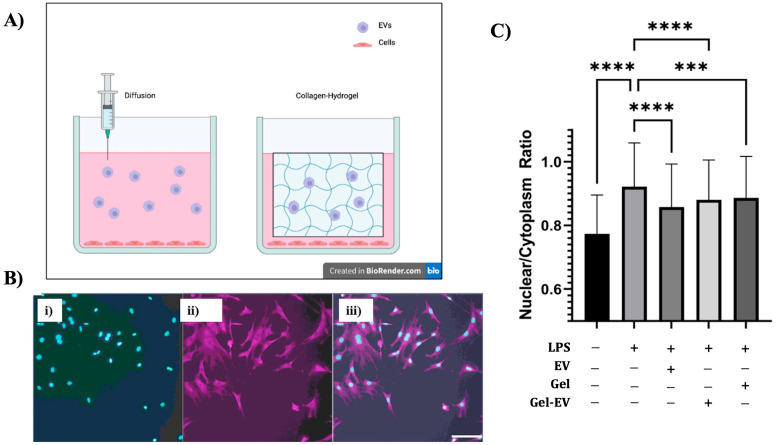
EV delivery in hydrogels to reduce LPS-induced inflammation. (**A**) Schematic illustration of hMSC EV delivery by direct addition (left) or encapsulated in collagen–hydrogels (right) to the LPS-stimulated cells. (**B**) Representative images of cells stained with DAPI (**i**) for cell nucleus, NF-kB (**ii**), and a superimposed image (**iii**) for collagen–hydrogel–EV delivery in the LPS-stimulated cells. Scale bar: 20 µm. (**C**) NF-kB ratio for expression in the nucleus versus cytoplasm for various treatment conditions. *** *p* < 0.001, **** *p* < 0.0001.

To facilitate EV delivery via hydrogels for inflammation reduction, 3T3 fibroblast cells were seeded on fibronectin-coated coverslips in culture plates. They were stimulated with LPS, and then introduced to EVs, collagen–hydrogel infused with EVs, and collagen–hydrogel not infused with EVs (Figure 7A). The localization of NF-kB inside nuclei was examined (Figure 7B). Based on the results (Figure 7C), it is clear to see the positive impact EVs have as anti-inflammatory agents. More ANOVA and *t*-tests were calculated, with extremely significant results—*p* < 0.001. The results confirmed LPS as an inflammatory agent that decreased in concentration via nucleus-to-cytoplasm ratio in all treatment groups. The group of EVs allowed to diffuse had a larger decrease in ratio compared to the inflammation-with-no-treatment group. Both collagen infused with EVs and collagen alone showed significant change when compared to inflammation with no treatment. However, the collagen infused with EVs seemed to have a stronger impact on the NF-kB ratio and was more significant in difference compared to the collagen hydrogel not infused with EVs. The results confirm the EVs’ anti-inflammatory properties with both the diffusion and collagen-infused hydrogel conditions.

## 4. Discussions

### 4.1. Influence of Dynamic Bioreactor Microenvironment on EV Secretion from hMSCs

Biomechanical stress has been recognized as a critical factor in regulating EV biogenesis [28]. In this study, hMSCs were preconditioned in vertical wheel bioreactors and compared to an SFB, static 2D planar culture, and static 3D aggregate culture with respect to EV secretion. Since hMSC expansion has been shown to be affected by a variety of factors such as tissue source and passage number [54], seeded cells were subcultured in a standard 2D condition, with morphologies constantly imaged to access viability and confirmed with flow cytometry. Further imaging of hMSCs seeded into the experimental groups showed cell expansion and growth in both bioreactor groups and the static 2D and aggregate controls. The difference in EV secretion due to different culture systems was assessed with nanoparticle tracking analysis, with both bioreactor samples showing significantly higher total EV production and EV productivity compared to the 2D control. Furthermore, increased EVs produced per media input and EVs produced per cell was shown in all bioreactor samples compared to the 2D group and the VWBR compared to the SFB. This increase in EV production from the static 2D culture to the dynamic bioreactors is consistent with our previous studies comparing bioreactor culture EV biogenesis to 2D culture [55,56]. Furthermore, determining gene expression through RT-qPCR, EV biogenesis in VWBR vs. static ULA showed upregulation of ESCRT-dependent and -independent gene expression for EV sorting and secretion in the VWBR group. This upregulation of genes correlates with the increase in EV generation per million cells that the VWBR had when compared to the ULA static culture. These results are supported by hypotheses that link the dynamic microenvironment to more efficient nutrient transfer, with more media exchange and waste removal. Furthermore, hMSCs are highly mechanosensitive, with cellular growth, differentiation, and EV biogenesis possibly being modulated by mechanical stimulation [57,58]. Thus, the dynamic microenvironment of the bioreactor systems may also stimulate cellular development, resulting in additional EV production. Additionally, dynamic bioreactor systems mimic in vivo cell growth in a more controlled culture setting [59], with biochemical signals that regulate hMSC growth and EV production [60,61,62,63,64].

However, the differences between bioreactors with respect to EV biogenesis have not been studied to the extent of differences between 2D and bioreactor culture. The consistent EV secretion increases in the VWBR compared to the SFB in both total EV produced and in productivity metrics in this study may be due to differences in shear stress. This is a significant factor in the effects of the microenvironment on hMSC cellular processes [65,66,67], with nuanced influences, such as contributing to differences in cell fate and phenotypic expression while still maintaining hMSC properties such as multi-lineage differentiation potential. However, specific effects on hMSC EV biogenesis by shear stress are not fully understood, with sparse literature explaining its effects on the EV biogenesis of bioreactor-cultured hMSCs. One explanation of shear stress’s effect on cell function is a possible buildup of ROS that may cause senescent behavior in cultured hMSCs, which has been shown to alter the hMSC secretome and may affect cell metabolism [55]. However, ROS effects on cell senescence are a gradual shift and may not be noticed in one bioreactor culture passage. An additional explanation of the effects of shear stress is the physical shearing of hMSC-EVs off the cell membrane by the dynamic culture, but this hypothesis was not reflected in this study, as the lower-shear-stress VWBR produced more EVs than the high-shear-stress SFB. In addition, it was found that around 50% of aggregate cells and up to 80% of microcarrier cells were exposed to shear stress, further contradicting the physical shearing hypothesis. Furthermore, in our previous study, it was shown that an increase in shear stress via agitation speed does little to affect hMSC EV biogenesis in the same bioreactor model. Bioreactor-related mechanical properties have also been shown to impact EV cargo loading profile and molecular composition [56]. These variations in protein profiles and cargo content, potentially modulated by culture conditions, have consistently been shown to improve therapeutic efficacy and possibly the longevity and storage of manufactured hMSC-EVs [68,69]. Increased EV biogenesis has also been consistently shown in various bioreactor models with differences in stretch and compression forces, in addition to shear stress (Supplementary Table S3). These increases have also been reported in cultures utilizing complex scaffold or organoid cultures; however, variations between systems make it difficult to isolate and evaluate these specific forces and their impacts [28]. Inconsistencies in how mechanical stresses affect the EV biogenesis of hMSCs may be due to differences in bioreactor model or aggregation, important factors that have been shown to influence cell processes in vitro and should be studied further for optimization of bioreactor systems for the development of hMSC EV-based therapeutics. Thus, with both a gap in the literature and heavy implications, future work examining such mechanical stresses as well as their specific effects on EV biogenesis, including EV production pathways, cargo loading, and cellular mechanisms, is highly warranted.

### 4.2. hMSC EV Functional Analysis

Quantification of isolated EVs was performed utilizing TEM and Western blotting for common exosomal markers to confirm that the PEGylated ultracentrifugation method was effective and consistent with our previous studies [44,70]. The anti-inflammatory properties of the isolated EVs were analyzed with an in vitro functional assay involving Aβ42 oligomer stimulation of healthy hMSCs to induce inflammation and high levels of ROS production. This stimulation promoted ROS production of the treated hMSCs; however, cells stimulated with both Aβ and EVs had ROS levels reduced to a level similar to the EV-only and no-treatment controls. This in vitro assay provides further evidence for the use of hMSC-EVs to combat neurodegenerative progression, which is often highlighted through high levels of ROS, which have been linked to apoptosis, decreases in cellular proliferation, and unwanted/unregulated immune response [71,72,73]. The literature is scarce on the specific cargo carried by EVs to produce this ROS reductive effect, and as a result, further research is needed to understand what cargo is carried and how more complex in vitro models involving different cell types and biomechanics similar to an in vivo setting would affect the efficiency of EV treatments. In addition, TNF-α expression decreased 4 days after Aβ42 and EV treatments and NF-kB nuclear localization was decreased after LPS and EV exposure. These results provide further evidence for the anti-inflammatory properties of EVs and potential use in combating inflammation during neurodegeneration. TNF-α promotes inflammatory responses in cells by activating other inflammatory cytokines, resulting in the attraction of macrophages and neutrophils to the site and further amplifying the inflammatory effect [74]. Understanding the mechanism behind EVs’ ability to lower TNF-α expression overtime is critical to developing complex models to maximize EV therapeutic effects.

Novel in vitro functional assays utilizing patient-specific AD media to generate neural degeneration models were used to verify the therapeutic effect of EVs. Common indicators of AD progression, such as high levels of ROS and inflammation, were present after 48 h of incubation with spent AD media. However, cells stimulated with both spent AD media and EVs had ROS levels reduced to a level similar to the EV-only group and lower than the no-treatment controls (by 39% and 31%, respectively). Inflammatory markers were upregulated once exposed to AD media. However, after EV incubation, significant downregulation of inflammatory markers provided further evidence of EVs’ ability to reduce inflammation and neural degeneration. Regarding anti-inflammatory markers, the condition that received only spent AD media had significantly higher gene expression with respect to the control as well as the +AD + EV condition. The phenomenon of upregulation of both pro- and anti-inflammatory markers in AD has been documented before [75]. A meta-analysis and systematic review on the correlation of pro- and anti-inflammatory markers such as TNF-α, IL6, IL10, and TGF-β1 in AD showed that upregulation of both pro- and anti-inflammatory markers can occur, indicating the complex interplay of biochemical pathways and AD progression on gene expression [76,77].

Along with understanding the cargo and pathways effected by EVs, another big hurdle that needs to be overcome for the efficient implementation of hMSC-EVs as a viable treatment is an effective method of delivery to treatment sites. This study shows that simple diffusion can allow for EVs to reach the treatment site, but in vivo there might be potential accumulation sites or routes of EV clearance that prevent EVs reaching injured tissue. However, a collagen-infused hydrogel with EVs embedded showed the most potential for drug delivery in the experimental model. It is unclear whether the concentration of EVs that arrived at the target sites varies regarding diffusion vs. collagen-infused hydrogel, so an EV labeling test is needed to confirm if this increased significance is due to more EVs arriving at the target site or due to the anti-inflammatory properties of collagen. In addition to that, dosage dependence and accumulation rate tests are needed to understand the kinetics of EV delivery for future in vivo testing.

## 5. Conclusions

This study demonstrates that the dynamic microenvironments of these bioreactors, characterized by their distinct fluid profiles, impact the EV secretion of hMSCs. Through EV isolation and NTA, both SFB and VWBR cultures showed an increase in EV secretion compared to the static 2D group, in total EVs produced, EVs per medium, and EVs per cell. Furthermore, the VWBR significantly increased EV production compared to static aggregate control. EV biogenesis gene expression was also promoted for VWBR compared to the static culture of hMSC aggregates. The EVs, delivered in medium or in hydrogels, reduced oxidative stress, inflammation cytokines, and NF-kB nuclear localization. Additionally, EVs were shown to reduce neural inflammation and degeneration in cells treated with conditioned media that model Alzheimer’s disease. This study shows that dynamic bioreactors are a viable method of hMSC preconditioning, producing a high amount of EV products and displaying efficacy in their usage. This study also suggests the promoted EV biogenesis of hMSCs in the VWBR may be linked to both ESCRT-dependent and independent-pathways. Additionally, this study reveals insights into how unique biophysical fluidic dynamics present in distinct culture systems directly impact EV biogenesis of adipose-derived hMSCs for potential preclinical and clinical applications in treating neurological disorders.

## Data Availability

The datasets generated during and/or analyzed during the current study are available from the corresponding authors on reasonable request.

## Supplementary Materials

The following supporting information can be downloaded at https://www.mdpi.com/article/10.3390/bioengineering12090933/s1. Table S1. A list of antibodies. Table S2. Primer information for RT-qPCR. Table S3. Mechanical Forces in Bioreactor Systems and Their Effects on EV Biogenesis. Figure S1: ROS production of hMSCs under various treatments with EVs and or Aβ42 oligomers with quantification. Scale bar: 50 µm. Figure S2: The standard curve for ELISA assay. Refs. [78,79].

## Abbreviations

The following abbreviations are used in this manuscript:

hMSCs	human mesenchymal stem cells
iPSCs	induced pluripotent stem cells
EVs	extracellular vesicles
ULA	ultralow attachment
SFB	spinner flask bioreactor
VWBR	vertical wheel bioreactor
FBS	fetal bovine serum
PBS	phosphate-buffered saline
NTA	nanoparticle tracking analysis
TEM	transmission electron microscopy
RT-qPCR	reverse-transcription quantitative polymerase chain reaction
NF-κB	nuclear factor kappa-light-chain-enhancer of activated b cells
ROS	reactive oxygen species
ESCRT	endosomal sorting complex required for transport
TNF-α	tumor necrosis factor
Aβ42	amyloid beta 1–42
YAP	Yes-associated protein
LPS	lipopolysaccharide
ANOVA	analysis of variance
