# Supplementary material for: Extracellular Vesicle Secretion from 3D Culture of Human Adipose-Derived Mesenchymal Stem Cells in Scalable Bioreactors
Source: Bioengineering (Basel). 2025 Aug 29;12(9):933. doi: 10.3390/bioengineering12090933 (PMC12467516; doi:10.3390/bioengineering12090933)
Supplement: Supplementary file 1 [file bioengineering-12-00933-s001.zip › bioengineering-3820622-supplementary.pdf]

Supplementary Materials

Extracellular Vesicle Secretion from 3D Culture of Human Adipose-derived Mesenchymal Stem Cells in Scalable Bioreactors

Shaoyang Ma[1,2,#], Justice Ene[1,#], Colton McGarraugh[1], Shaoxuan Ma[1,3], Colin Esmonde[1], Yuan Liu[1,*], Yan Li[1,*]

1. Department of Chemical and Biomedical Engineering, FAMU-FSU College of Engineering, 2525 Pottsdamer St., Tallahassee, FL 32310, USA
2. Brown University, 69 Brown St., Providence, RI.
3. Lawton Chiles High School, 7200 Lawton Chiles Ln, Tallahassee, FL 32312, USA

[#]These two authors contributed equally to this work.

Supplementary Table S1. A list of antibodies.

**Primary Antibody**	**Origin/ Isotype**	**Supplier/Cat#**	**Dilution**
YAP	Rabbit IgG	Santa Cruz, sc-15407	Flow Cytometry: 1:200
Sirt-1	mouse monoclonal IgG_1	Santa Cruz/sc-74465	Western blot: 1:1000<br>Flow Cytometry: 1:200
NF-KB P65	Rabbit monoclonal IgG	Cell Signaling Technology, 8242S	Immunocytochemistry: 1:400
Calnexin	Rabbit IgG	Cell Signaling Technology, 2433	Western blot: 1:1000
CD81	Rabbit IgG	Cell Signaling Technology, 56039	Western blot: 1:1000
HSC70	Rabbit IgG	Cell Signaling Technology, 8444	Western blot: 1:1000
TSG101	Mouse IgG_1	Santa Cruz, sc-136111	Western blot: 1:1000
**Secondary Antibody**	**Origin/ Isotype**	**Supplier/Cat#**	**Dilution**
Alexa Fluor™ 488	Goat anti-Rabbit IgG (H + L)	Invitrogen, A-11008	Immunocytochemistry and Flow cytometry: 1:400
Alexa Fluor™ 488	Goat anti-Mouse IgG_1	Invitrogen, A-21121	Immunocytochemistry and Flow cytometry: 1:400
Alexa Fluor™ 647	Goat anti-Rabbit IgG (H + L)	Invitrogen, A-21245	Immunocytochemistry: 1:1000
IRDye® 800CW	Goat anti-mouse IgG_1	LI-COR, 926-32350	Western blot: 1:5,000
IRDye® 800CW	Goat anti-Rabbit IgG (H + L)	LI-COR, 926-32211	Western blot: 1:5,000

Supplementary Table S2. Primer information for RT-qPCR.

Primer number	Gene name	Primer name in database	Primer sequence (5'-3')
1	ACTB	Bactin F	GTACTCCGTGTGGATCGGCG
		Bactin R	AAGCATTTGCGGTGGACGATGG
2	CD63	Forward	ACAACCACACTGCTTCGATCC
		Reverse	GACTCGGTTCTTCGACATGGA
3	SMPD2	SMPD2-F1	GCCTGGGAGACTTTCTGAACC
		SMPD2-R1	AAGTGGTGTGCAGCTGGGTAG
4	SRSF5(hrs)-1	SRSF5-F1	CTTCTCGGATCGAGGCTTCTT
		SRSF5-R1	TCGAATCAACTGCGCTCATTA
5	TSG101	TSG101 F	CACCTGGTGGTCCATATCCTG
		TSG101 R	GATGGTGTCCTCGCTGATTGT
6	STAM1	STAM1-F1	CACTGGATTTTTGGGTTGCTC
		STAM1-R1	GTGGAAAACATTTTTCGCATGA
7	PDCD61P (ALIX)	PDCD61P F	TAAGTGCATCTGAGGGCCAAA
		PDCD61P R	GGGGCCTCCTTTCCTAGTTTC
8	MITF-1	MITF-F1	GAATTGGTGATGGGTGATGGA
		MITF-R1	TGCATGGGAACTATGCAGTTG
9	RAB27B	RAB27B F	TCCATGAAGCTGCTTGTCTCA
		RAB27B R	GTTGGGTCTCCACCCAGAAAT
10	TNFa	TNFa F	TGAGGAGGACGAACATCCAAC
		TNFa R	ACCCTAAGCCCCCAATTCTCT
11	IL6	IL6 F	ACCCCCAATAAATATAGGACTGGA
		IL6 R	GGAGTTCATAGCTGGGCTCCT
12	IL12B	IL12B F	CTGAGGAGAGTCTGCCCATTG
		IL12B R	GTTCTTGGGTGGGTCAGGTTT
13	MAPT	MAPT-F	GCTGAGATCACTCGCTTCACC
		MAPT-R	CTGTAGATGGGACACGCAGGT
14	BACE-1	BACE1-F	TTTGGCTTCAAAGGATTTTGG
		BACE1-R	CTTAGGCCACCTGCCGTATTT
15	APP	APP-F	CGAAGTTGAGCCTGTTGATGC
		APP-R	CGGAATTCTGCATCCATCTTC
16	CD163	CD163 F	CCTCGTGCAATTCATCGTCTT
		CD163 R	CACAGCGACCTCCTCCATTTA
17	IL10	IL10 F	TGGGGCTTCCTAACTGCTACA
		IL10 R	GAAGTGGTTGGGGAATGAGGT
18	TGFB`	TGFB1 F	GAAGAACTGCTGCGTGCGGC
		TGFB1 R	CTGCGTGTCCAGGCTCCAAATG
19	P53	P53 F1	CTGGACGACAGGCAGACTTT
		P53 R1	GCACAAACACGAACCTCAAA

Supplementary Table S3. Mechanical Forces in Bioreactor Systems and Their Effects on EV Biogenesis

Bioreactor	Cell Type	Mechanical Force	EV Outcome	Additional Notes
Vertical Wheel Bioreactor [1]	iPSC	Low levels of shear stress at 0.1-0.3 dyn/cm^2	Increased EV secretion (17-23 fold); differing protein and miRNA cargo	EVs from VWBR had the highest quality and quantity
Spinner Flask Bioreactor [2]	hMSC	Shear stress 50, 80 RPM (3, 6 dyn/cm^2)	Increased EV secretion (40%); increased ROS	ROS did not significantly impact senescence
Scaffold-Perfusion Bioreactor [3]	hMSCs	Flow-derived shear stress at 1, 5, or 10 mL/min	Increased EV secretion (40-80 fold); improved wound healing	Maximized EV production at 5mL/min flow rate
Hollow-fiber Bioreactor [4]	hMSCs	(Flow rate: 22-28 times/min)	Increased exosome secretion (up to 19.4-fold); improved anti-inflammatory effect	Higher exosome collection efficiency in bioreactor
Flat Plate Bioreactor [5]	hMSCs	Shear stress at 0.1-1mL/min (1.0*10^-4 – 1.0*10^-3 dyn/cm^2)	Increased EV secretion (7 fold); improved therapeutic potential	EVs resulted in tissue regeneration in acute kidney injury model

Supplementary Figure S1: ROS production of hMSCs under various treatments with EVs and or Aβ42 oligomers with quantification. Scale bar: 50 μm.

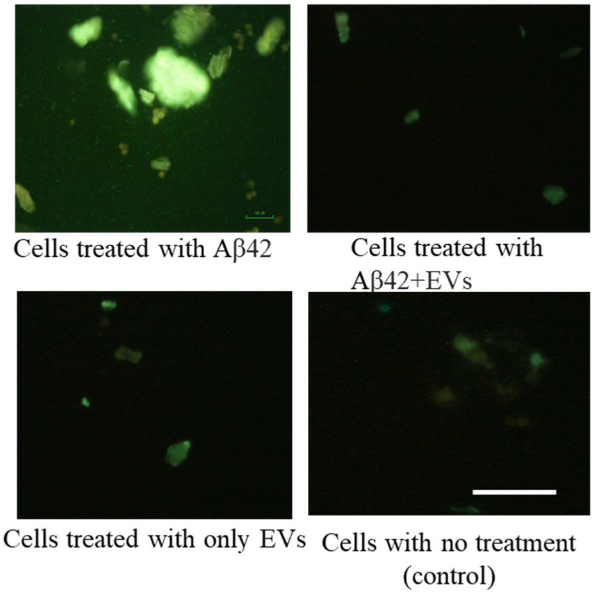

	Mean	S_x
Aβ+	122.740	34.984
Aβ+EV+	70.248	29.316
EV+	73.691	33.917
Control	50.805	31.821

Supplementary Figure S2: The standard curve for ELISA assay.

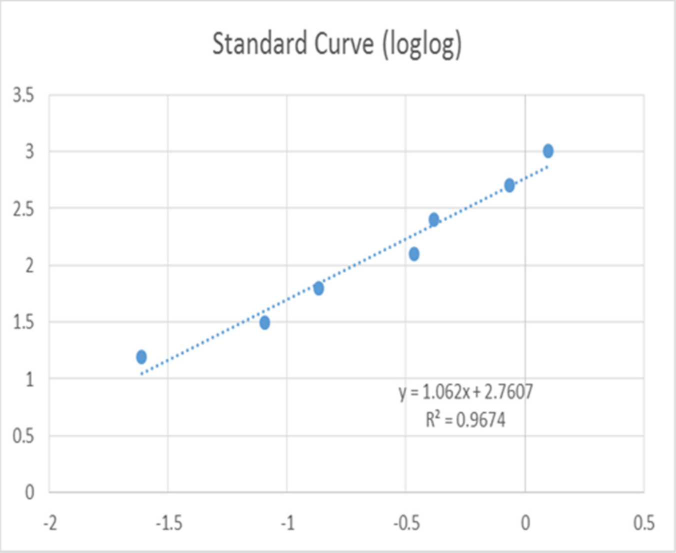

References

[1] L. Muok *et al.*, "Extracellular vesicle biogenesis of three-dimensional human pluripotent stem cells in a novel Vertical-Wheel bioreactor," *J Extracell Biol,* vol. 3, no. 1, p. e133, Jan 2024, doi: 10.1002/jex2.133.

[2] R. Jeske, X. Chen, S. Ma, E. Z. Zeng, T. Driscoll, and Y. Li, "Bioreactor Expansion Reconfigures Metabolism and Extracellular Vesicle Biogenesis of Human Adipose-derived Stem Cells In Vitro," *Biochem Eng J,* vol. 188, Dec 15 2022, doi: 10.1016/j.bej.2022.108711.

[3] S. M. Kronstadt *et al.*, "Mesenchymal Stem Cell Culture within Perfusion Bioreactors Incorporating 3D-Printed Scaffolds Enables Improved Extracellular Vesicle Yield with Preserved Bioactivity," *Adv Healthc Mater,* vol. 12, no. 20, p. e2300584, Aug 2023, doi: 10.1002/adhm.202300584.

[4] J. Cao *et al.*, "Three-dimensional culture of MSCs produces exosomes with improved yield and enhanced therapeutic efficacy for cisplatin-induced acute kidney injury," *Stem Cell Res Ther,* vol. 11, no. 1, p. 206, May 27 2020, doi: 10.1186/s13287-020-01719-2.

[5] H. Kang, Y.-h. Bae, Y. Kwon, S. Kim, and J. Park, "Extracellular Vesicles Generated Using Bioreactors and their Therapeutic Effect on the Acute Kidney Injury Model," *Advanced Healthcare Materials,* vol. 11, no. 4, p. 2101606, 2022/02/01 2022, doi: https://doi.org/10.1002/adhm.202101606.
